# Patterns of Somatic Variants in Colorectal Adenoma and Carcinoma Tissue and Matched Plasma Samples from the Hungarian Oncogenome Program

**DOI:** 10.3390/cancers15030907

**Published:** 2023-01-31

**Authors:** Alexandra Kalmár, Orsolya Galamb, Gitta Szabó, Orsolya Pipek, Anna Medgyes-Horváth, Barbara K. Barták, Zsófia B. Nagy, Krisztina A. Szigeti, Sára Zsigrai, István Csabai, Péter Igaz, Béla Molnár, István Takács

**Affiliations:** 1 Department of Internal Medicine and Oncology, Faculty of Medicine, Semmelweis University, 1083 Budapest, Hungary; 2 MTA-SE Molecular Medicine Research Group, Eötvös Loránd Research Network, 1083 Budapest, Hungary; 3 Department of Physics of Complex Systems, ELTE Eötvös Loránd University, 1117 Budapest, Hungary; 4 Department of Endocrinology, Faculty of Medicine, Semmelweis University, 1083 Budapest, Hungary

**Keywords:** colorectal adenoma, colorectal carcinoma, CRC, tissue biopsy, liquid biopsy, whole-exome sequencing

## Abstract

**Simple Summary:**

Colorectal cancer is a highly lethal cancer type with a high incidence and mortality rate in Hungary. To explore the genetic background behind this epidemiological challenge, an emerging number of studies have aimed to explore colorectal carcinomas, but less is known about adenomas; therefore, we aimed to analyze tissue biopsies from both sample types in a comprehensive way by whole-exome sequencing (WES). As liquid biopsy has certain advantages over tissue sampling, we included matched plasma-originated cfDNA samples and examined the differences between colorectal cancer and adenomas by WES and targeted sequencing. According to our WES results, a high correlation was found between matched tissue and plasma variant allele frequencies. Liquid biopsy is a suitable starting material for WES and also for targeted panel sequencing, with the latter providing higher coverage depth; therefore, plasma-derived cfDNA may gradually become the first choice for genetic characterization of CRC patients in the future.

**Abstract:**

Analysis of circulating cell-free DNA (cfDNA) of colorectal adenoma (AD) and cancer (CRC) patients provides a minimally invasive approach that is able to explore genetic alterations. It is unknown whether there are specific genetic variants that could explain the high prevalence of CRC in Hungary. Whole-exome sequencing (WES) was performed on colon tissues (27 AD, 51 CRC) and matched cfDNAs (17 AD, 33 CRC); furthermore, targeted panel sequencing was performed on a subset of cfDNA samples. The most frequently mutated genes were *APC*, *KRAS*, and *FBN3* in AD, while *APC*, *TP53*, *TTN*, and *KRAS* were the most frequently mutated in CRC tissue. Variants in *KRAS* codons 12 (AD: 8/27, CRC: 11/51 (0.216)) and 13 (CRC: 3/51 (0.06)) were the most frequent in our sample set, with G12V (5/27) dominance in ADs and G12D (5/51 (0.098)) in CRCs. In terms of the cfDNA WES results, tumor somatic variants were found in 6/33 of CRC cases. Panel sequencing revealed somatic variants in 8 out of the 12 enrolled patients, identifying 12/20 tumor somatic variants falling on its targeted regions, while WES recovered only 20% in the respective regions in cfDNA of the same patients. In liquid biopsy analyses, WES is less efficient compared to the targeted panel sequencing with a higher coverage depth that can hold a relevant clinical potential to be applied in everyday practice in the future.

## 1. Introduction

Colorectal cancer (CRC) has a continuously increasing incidence and mortality with an estimated 1.9 million new cases and 900,000 registered deaths per year worldwide in 2020 [1]. The outstanding importance of CRC is also shown among all cancer types as it stands at 9.4% in men and 10.1% in women [2]. CRC is one of the most curable cancers if detected in early stages; therefore, nationwide screening programs are gaining increased significance with a high potential to improve the current statistics [3,4]. In parallel, molecular markers of CRC are of emerging importance as they are crucial in the early detection of the disease and help in the objective classification and the successful management of CRC patients [2,5]. Interestingly, geographic and ethnic background-related differences are known to be characteristic of CRC cases [6], e.g., incidence differences can be detected in Westernized countries vs. all other countries [2]. Eastern Europe, especially Hungary, has one of the highest CRC mortality and incidence rates [1,7]. In the 40 years between 1975 and 2014, CRC incidence increased by 62%, reaching 10,684 cases in 2013, when a total of 5017 colorectal-cancer-related deaths were registered in Hungary [8]. This relatively high number is evident not only on a national scale but also on a worldwide scale. According to a study by Xi et al., in 2020, Hungary had the highest age-standardized incidence rate with 45.3 cases per 100,000 persons [1]. On the other hand, therapy response and survival rates are also not favorable in the Hungarian population [7], e.g., the 5-year overall survival (OS) probability of CRC appears to be worse than certain international references [9]. Almost two-thirds of the Hungarian CRC cases are distally located with higher frequency in men [6]. Certain parameters that fundamentally influence gastrointestinal tumor development, such as the level of physical activity and dietetic and environmental factors, might also be responsible for outstanding CRC incidence [10,11].

According to the classical model, early benign adenomas can develop into a malignant CRC by the continuous accumulation of genetic mutations leading to a characteristic mutation profile of each stage [12]. The well-known CRC-specific markers, such as *APC*, *KRAS*, *BRAF*, *HER2*, and *TP53* somatic mutations and the microsatellite stability status, are also critical factors for tumor classification and optimal therapy decision [13,14,15]. Whole-exome sequencing can be an ideal approach to identify variants characteristic to a certain region or ethnicity. In parallel, a continuously growing number of databases, such as the colon and rectal adenocarcinoma project of The Cancer Genome Atlas (TCGA-COAD, READ), are widely used to compare gene mutation frequencies in a certain population to international references [16,17].

Moreover, analysis of the plasma circulating cell-free DNA (cfDNA) has increasing importance in the diagnosis and the monitoring of CRC progression as it can serve as a highly sensitive, rapid, yet minimally invasive tool in the hands of researchers and clinicians [18]. It can be a relevant alternative for tissue biopsies, primarily because it can overcome the intratumoral-heterogeneity-originated sampling errors [19]. Besides the well-known, frequently mutated, and clinically relevant genes in CRC (e.g., *KRAS*, *BRAF*, *APC*, and *PIK3CA*), the identification of novel variants by WES offers a possibility to expand our knowledge about CRC formation and assess the region/country-specific variants. Recently, in parallel with the emerging importance of NGS-based cfDNA marker discovery studies, targeted panel assemblies focusing on a few to hundreds of frequently mutated target sites became commercially available for focused diagnosis, therapy selection, and prognostic analyses.

The present study aimed to shed light on the comprehensive picture of the genetic variants in the exome characteristic of Hungarian patients with colorectal tumors. As liquid biopsy has certain advantages over tissue sampling, we included matched plasma-originated cfDNA samples in our study to examine the differences between colorectal cancer and adenomas by high coverage exome sequencing in both tissue and plasma samples. The analysis of cfDNA was supplemented with deep-sequencing using a CRC-focused targeted panel analysis.

## 2. Materials and Methods

### 2.1. Clinical Samples

In the framework of the Hungarian Oncogenome Program (https://cbioportal.vo.elte.hu/cbioportal/; accessed on 19 September 2022), 172 colonic and rectal tissue (18 normal samples of healthy individuals, 76 normal adjacent tissue, 27 adenomas, and 51 CRC) and 35 buffy coat samples (15 of healthy controls (NEG) and 10–10 from patients with adenoma or CRC, respectively) were involved in our whole-exome sequencing study. Samples were obtained after the written informed consent of untreated patients. Colonic biopsy specimens were collected during the endoscopic intervention, and surgically removed colon or rectum tissue samples were obtained from tumors and histologically normal adjacent tissue (NAT) before treatment at the 2nd Dept of Internal Medicine and the 1st Dept of Surgery, Semmelweis University, Budapest, Hungary. Samples were then stored in RNALater Stabilization Solution (ThermoFisher Scientific, Waltham, MA, USA) at –80 °C until use. Moreover, tissue samples from the same sites were immediately fixed in buffered formalin, and experienced pathologists established histological diagnoses. The detailed patient specification with age, sex, anatomic location, histology, and AJCC stage [20] data is described in Table 1. Blood samples were collected directly before the diagnostic examinations or surgery (7 NEG, 17 AD, 33 CRC), and plasma fraction was separated within 4 h by double centrifugation at 1350× *g* for 12 min followed by buffy coat collection. The study was conducted according to the Declaration of Helsinki and approved by the local ethics committee and government authorities (Regional and Institutional Committee of Science and Research Ethics (ETT TUKEB) Nr.: 14383-2/2017/EKU Semmelweis University, Budapest, Hungary). MSI status was determined by MSIsensor2 (https://github.com/niu-lab/msisensor2 accessed on 27 January 2020) and was validated by immunohistochemistry analyses.

### 2.2. DNA Isolation

Tissue samples were homogenized in Tissue Lysis Buffer using the MagNA Lyser instrument with the MagNA Lyser Green Beads Tubes (Roche Diagnostics GmbH, Manheim, Germany). Samples were digested with 4 mg/µL proteinase K (Roche Diagnostics GmbH) at 56 °C for 2 h and 1 h in the case of tissue and buffy coat samples, respectively. Genomic DNA was isolated using the High Pure PCR Template Preparation Kit (Roche Diagnostics GmbH) according to the manufacturer’s instructions. The RNA content of the samples was eliminated with the RNase A/T1 Mix (2 mg/mL of RNase A and 5000 U/mL of RNase T1, ThermoFisher Scientific, Vilnius, Lithuania) for 1 h at 37 °C. Genomic DNA was eluted in 100 µL RNase- and DNase-free water and stored at −20 °C until use. The concentration of dsDNA was determined using a Qubit 1.0 fluorometer with the Qubit dsDNA HS Assay Kit (Invitrogen, Waltham, MA, USA).

CfDNA was isolated with the Quick-cfDNA Serum and Plasma Kit (Zymo Research Corp, Irvine, CA, USA) from 3–5 mL plasma per patient. CfDNA was quality assessed by BioAnalyzer 2100 microcapillary electrophoresis system (Agilent Technologies, Santa Clara, CA, USA) and was quantified by the HS dsDNA Assay Kit with a Qubit 1.0 instrument (Invitrogen).

### 2.3. Library Preparation and Next-Generation Sequencing

Library preparation of tissue-originated DNA samples was performed using the Nextera DNA Exome kit (Illumina Inc., San Diego, CA, USA) according to the manufacturer’s instructions. Briefly, 50 ng gDNA was tagmented using the Nextera transposome in a 50 µL volume at 58 °C for 10 min. Then, DNA fragments were PCR-amplified (10 cycles) with Index 1 and Index 2 adapters and sequences required for cluster amplification. The amplified libraries were purified using magnetic Samples Purification Beads (Illumina Inc.). The quantification of the purified libraries was carried out by a Qubit 1.0 fluorometer using the Qubit dsDNA HS Assay Kit (ThermoFisher). Fragment size distributions were analyzed using a BioAnalyzer capillary gel electrophoresis system with Agilent High Sensitivity DNA Chips and a High Sensitivity DNA Kit (Agilent Technologies). Nine amplified dsDNA libraries (500 ng each) per sequencing reaction were pooled and hybridized to Nextera DNA Exome Coding Exome Oligo capture probes (Illumina Inc.). The captured exome library pools were purified using Nextera DNA Exome Streptavidin Magnetic Beads (Illumina Inc.). In order to provide high specificity of the captured regions, repeated enrichment (second hybridization with Coding Exome Oligos and second capture with Streptavidin Magnetic Beads) was performed. After purification with magnetic Samples Purification Beads, the enriched library pools were amplified in 10-cycle PCR reactions using a Nextera DNA Exome PCR Primer Cocktail and Enrichment Amplification Mix (Illumina Inc.).

CfDNA WES libraries were performed using the QIASeq Human Exome Kit (Qiagen GmbH, Hilden, Germany) with 10–50 ng cfDNA input. After end-polish, adapter ligation, and amplification, samples were pooled equimolarly, and pools were subsequently hybrid captured, amplified, and purified. On the other hand, panel sequencing was also performed for 11 CRC patients whose cfDNA quantity was enough for this analysis in parallel to achieve a higher coverage of the CRC development-associated genes. QIAseq Targeted DNA Ultra for cfDNA (Qiagen GmbH) was applied as a solution for ultrasensitive targeted next-generation sequencing of cfDNA with hotspot coverage of *AKT1*, *APC*, *BRAF*, *CTNNB1*, *DPYD*, *EGFR*, *FBXW7*, *GNAS*, *MAP2K1*, *NRAS*, *PIK3CA*, *RET*, *SMAD4*, and *UGT1A1*, and with full exon coverage of *ERBB2*, *KRAS*, and *TP53*. After end-repair and A-addition, target enrichment was performed followed by universal PCR amplification and clean-up steps according to the manufacturer’s instructions. The purified whole-exome library pools were quantified using the KAPA Library Quantification Kit (KAPA Biosystems, Wilmington, MA, USA) and the QIASeq Library Quant Assay (Qiagen GmbH) in the case of cfDNA libraries. For quality testing, a Bioanalyzer 2100 system with Agilent High Sensitivity DNA Chips and a High Sensitivity DNA Kit (Agilent Technologies) were applied. Paired-end sequencing (2 × 150 or 2 × 75 cycles) was carried out on a NextSeq 500 device using a NextSeq 500/550 High Output Flow Cell Cartridge v2 and a NextSeq 500/550 High Output Reagent Cartridge v2 (300 cycles/150 cycles) (Illumina Inc.).

### 2.4. Bioinformatic Analyses

Demultiplexing and FASTQ file generation were performed using the Illumina BaseSpace interface. We employed the FastQC and MultiQC tools to assess the quality of sequencing reads. Raw sequence reads were aligned to the GRCh38 human reference genome using the Burrows–Wheeler Alignment Tool (BWA) and the BWA-MEM algorithm [26]. SNP and short indel germline and somatic variants were determined with the Genome Analysis Tool Kit (GATK) [27] pipeline version 4.1.4.1 according to the “Best Practices Workflows”, as described at https://gatk.broadinstitute.org/ (accessed on 18 September 2020). To enhance the filtering of false-positive somatic calls, we created a panel of normals (PoN) file by employing the MuTect2 algorithm of GATK in “tumor-only” mode, and all normal samples including those of healthy patients and normal adjacent tissue and buffy coat samples of adenoma and CRC patients as inputs. Variants present in at least two samples were included in the PoN file. Somatic mutations were identified by Mutect2 using the PoN file and tumor and paired normal (normal adjacent tissue or—if not available—buffy coat) samples as inputs simultaneously. Germline variants of each normal tissue and buffy coat sample were identified using the HaplotypeCaller algorithm of GATK [28]. In each case, variants were filtered with the default settings, and variant coordinates were transformed to the GRCh37 reference genome with CrossMap [29]. Variant files were annotated using the vcf2maf tool (Cyriac Kandoth. mskcc/vcf2maf: vcf2maf v1.6.19. (2020). doi:10.5281/zenodo.593251) and the Ensembl Variant Effect Predictor (VEP) release 94 [30]. Clinical impact of the variants was evaluated according to the ClinVar [31], dbSNP [32], COSMIC, and OncoKB databases [33]. Non-silent exonic and splice site somatic variants were evaluated. Somatic variation data were summarized, and the results were plotted by the maftools v2.8.05 R package [34].

We calculated tumor mutation burden values using the “tmb” function of the maftools program package as the number of non-silent mutations per Mb in samples of each data set. The target capture size was set to 45.3 Mb, according to the exome sequencing kit used for our samples, and we used 38 Mb as an estimate of the exome size for the reference data. TMB was compared between our cohort (SE-AD, SE-CRC), COCA, and TCGA groups by the Kruskal–Wallis test and subsequent pairwise comparisons with Wilcoxon rank sum tests and the Benjamini–Hochberg *p*-value adjustment. TMB values were plotted on a log10 scale.

The concatenated TCGA COAD + READ dataset (https://portal.gdc.cancer.gov/; accessed on 10 November 2019), and furthermore, somatic mutation data of CRC cases from a Chinese cohort (COCA), were used as reference datasets (accessed on 17 November 2021).

In order to determine genes with significantly different mutational frequencies between the analyzed datasets, Fisher’s exact tests were computed by using the “mafCompare” tool of the maftools program package in R.

Mutational signature analysis was performed for the AD and CRC tissue sample data and the TCGA dataset on the basis of the COSMIC Single Base Substitution signatures (v3.2—March 2021, https://cancer.sanger.ac.uk/signatures/sbs/; accessed on 4 March 2022) by using maftools.

### 2.5. Validation of KRAS Mutation Status Using Digital PCR Technology

As a technical validation of WES, the most abundant KRAS variant in our CRC samples, the G12D variant, was assessed with droplet digital PCR (ddPCR). The PCR reaction contained 11 µL ddPCR Supermix for Probes (2×, no dUTP) (Bio-Rad Laboratories Inc., Hercules, CA, USA), 1.1 µL multiplex primers/probes (wild-type alleles labeled with HEX, mutated alleles detected with FAM) (Bio-Rad), and 9.9 µL (50 ng) template DNA. After automated droplet generation using the QX200 AutoDG system (Bio-Rad), PCR amplification was carried out with the following thermocycling conditions: denaturation at 95 °C for 10 min, amplification for 45 cycles at 94 °C for 30 s, annealing at 55 °C for 1 min, followed by enzyme deactivation at 98 °C for 10 min, and 4 °C hold. Finally, droplets were detected with a QX200 Droplet Reader (Bio-Rad), and the results were analyzed with QuantaSoft Software v1.7 (Bio-Rad) to determine the ratio of mutated and wild-type alleles in the samples.

### 2.6. In Situ Hybridization

In situ hybridization (ISH) validation of *KRAS* G12D (c.35G > A) mutation experiments were performed on 5 µm thick formalin-fixed, paraffin-embedded (FFPE) tissue sections using the BaseScope technology (Advanced Cell Diagnostics Inc., Newark, CA, USA) [35]. The following BaseScope probes were incubated on tissue sections according to the manufacturer’s instructions: *KRAS* G12D (1 zz pair, cat no. 705519), dapB-negative control probe (a Bacillus subtilis gene, 414–862, 10 zz pairs, cat no. 701029), and PPIB-positive control probe (Cyclophilin B, 139–989, 16 zz pairs, cat no. 701049) (ACD Inc., Benton, AR, USA). Detection was performed using the horseradish peroxidase (HRP) kit and Discovery-rhodamine substrate (Roche). The CK AE1/3 mouse monoclonal antibody (1:200, Dako-Agilent, Glostrup, Denmark) was applied with Alexa-488-conjugated anti-mouse Ig (Jackson Immunoresearch, West Grove, PA, USA) for cytokeratin detection. Nuclei were stained with DAPI (ThermoFisher). Stained slides were scanned with the Pannoramic Confocal (3DHISTECH Ltd., Budapest, Hungary) digital slide scanner using a 40× objective and were examined with CaseViewer software v2.3 (3DHISTECH Ltd.).

## 3. Results

### 3.1. Whole-Exome Sequencing Parameters of Tissue Samples

**Figure 1:**
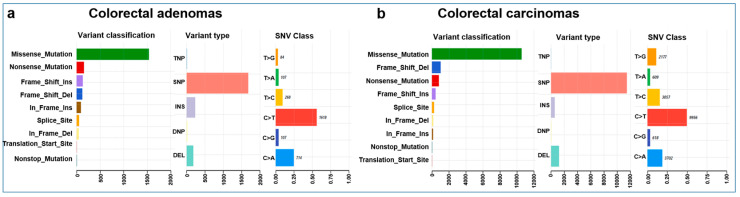
Summary plots of the variants found in colorectal adenomas (**a**) and carcinomas (**b**). Variant classification distribution: the *X*-axis represents the number of variants, and the *Y*-axis represents the variant type categories. Variant type plot: the *X*-axis represents the number of variants, and the *Y*-axis represents the variant type categories and SNV class plot.

The mean coverage of WES was 96× with a range of 30–159×. The total number of somatic mutations excluding mtDNA variants ranged from 18 to 465 in AD, with 31 to 6793 and 287 to 2422 in MSS and MSI CRC samples, respectively. The mean somatic mutation rate was 2.97 in AD, 7.68 in MSS, and 35.64 mutations/Mb in MSI CRC cases. AD and CRC cases showed significant differences in the overall somatic mutation number (Mann–Whitney–Wilcoxon test W = 302.5, *p* = 0.004). Given the low number (5/51) of MSI tumors in our cohort, MSS and MSI CRC cases were further analyzed together. Most of the non-silent variants were missense mutations, both in the AD and in the CRC groups. These were followed by nonsense mutations, frameshift insertions, and deletions in Ads, and frameshift deletions, nonsense mutations, and frameshift insertions in CRCs. Among the variant types, SNPs were dominant, followed by indels in both groups. Most of the observed SNVs were C > T, followed by C > A and T > C (Figure 1).

### 3.2. COSMIC Mutation Signatures

**Figure 2:**
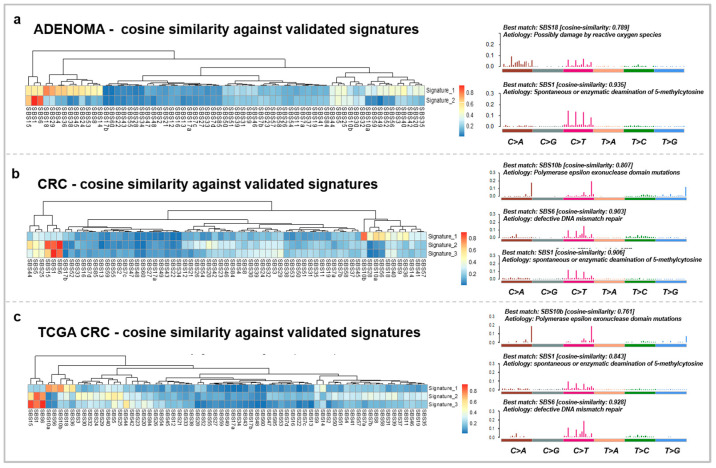
Somatic mutation signatures of the adenomas (**a**) and carcinomas of our cohort (**b**) and the TCGA COAD + READ CRC (**c**) dataset. The left panels represent the signatures retrieved from the analyzed groups and their similarity to the COSMIC v3.2 SBS signatures on heatmaps. The right panels show the characteristic signatures of the datasets and the proposed etiology of the SBS signatures.

Mutational signatures underlying AD and CRC from the present cohort were also assessed. In case of the AD group, two signatures were identified, showing the highest cosine similarities (CS) to single base substitutions 1 (SBS1) (CS: 0.935, etiology: spontaneous or enzymatic deamination of 5-methylcytosine) and SBS18 (CS: 0.789, etiology: possibly damage by reactive oxygen species) (Figure 2a). On the other hand, our CRC cases showed three distinct signatures, which were the most similar to SBS1 (CS: 0.906), SBS6 (CS: 0.903, etiology: defective DNA mismatch repair), and SBS10b (CS: 0.807, etiology: polymerase epsilon exonuclease domain mutations) (Figure 2b). In comparison, the same signatures were retrieved in our CRC cohort as in the TCGA COAD + READ dataset: SBS1 (CS: 0.843), SBS6 (CS: 0.928), and SBS10b (CS: 0.761) (Figure 2c).

### 3.3. Tumor Mutation Burden Evaluation

**Figure 3:**
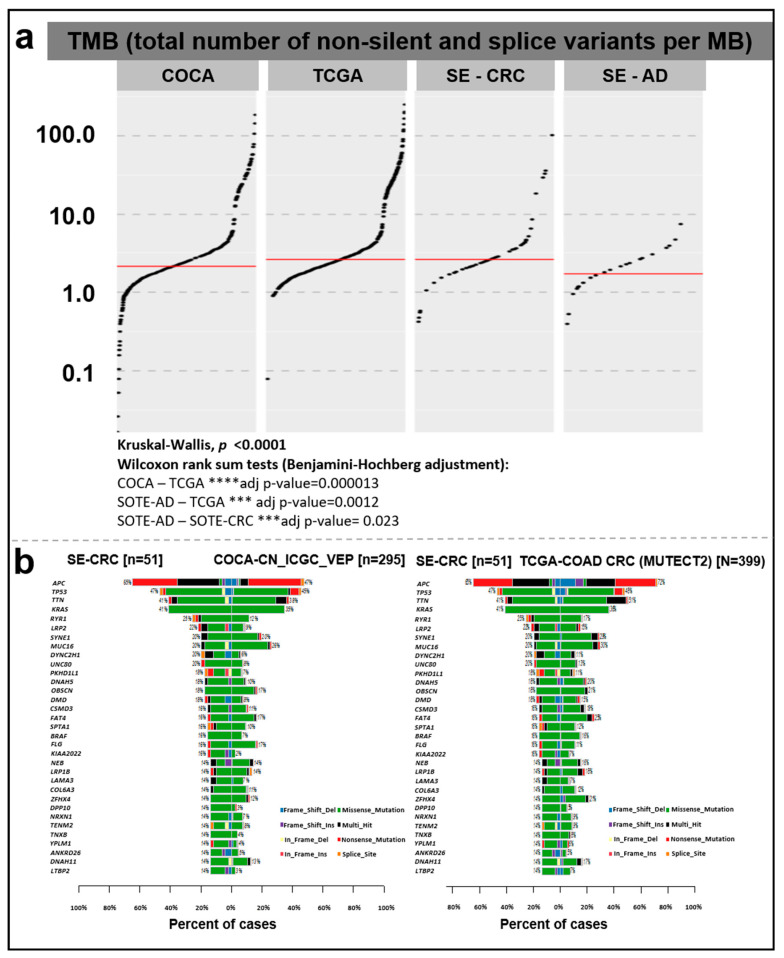
(**a**) Tumor mutation burden in AD and CRC samples of our cohort (SE-AD, SE-CRC) compared to the COCA and TCGA COAD + READ reference datasets. The total numbers of non-silent and splice site variants were divided by 38 Mb and were represented on a log10 Y-scale. The red lines show median values. (**b**) Direct pairwise comparison of mutation frequencies of genes in our cohort compared to COCA (left) and TCGA COAD + READ (right) datasets showing the percent of affected samples (%).

There was a significant difference in TMB between the COCA–TCGA, SE-AD–TCGA, and SE-AD–SE-CRC groups. In our cohort, CRC samples showed a relatively higher somatic mutation rate (median = 2.605 total/Mb) compared to AD tissue samples (median = 1.71 total/Mb) (Figure 3).

In the comparison of the different cohorts, the analyzed CRC group showed a higher median TMB rate (2.605 total/Mb) than the Chinese dataset (COCA, 2.13 total/Mb), while the concatenated TCGA colorectal cancer and rectal cancer (TCGA_COAD_READ; 2.605 total/Mb) datasets showed the same value (Figure 3a). According to the Kruskal–Wallis test (*p* < 0.0001) and subsequent pairwise comparisons with Wilcoxon rank sum tests, a significant difference was detected in TMB between the COCA–TCGA (adjusted *p*-value = 0.000013), SE-AD–TCGA (adjusted *p*-value = 0.0012), and SE-AD–SE-CRC groups (adjusted *p*-value = 0.023). Comparing our CRC data with the Chinese reference (COCA), a relatively higher mutation rate was detected (with a minimum of 5% difference), e.g., in the case of *APC*, *KRAS*, *RYR1*, *LRP2*, *DYNC2H1*, *UNC80*, *PKHD1L1*, *DNAH5*, *DMD*, *CSMD3*, *SPTA1*, *BRAF*, *KIAA2022*, *LAMA3*, *DPP10*, *NRXN1*, *TENM2*, *TNXB*, *YLPM1*, *ANKRD26*, and *LTBP2*, while in the case of *MUC16*, a lower percentage of cases was found among our CRC samples. In a similar comparison with the TCGA COAD dataset, the *KRAS*, *RYR1*, *LRP2*, *DYNC2H1*, *UNC80*, *PKHD1L1*, *FLG*, *KIAA2022*, *LAMA3*, *DPP10*, *NRXN1*, *TENM2*, *TNXB*, *YLPM1*, *ANKRD26*, and *LTBP2* genes showed a higher, while the *APC*, *TTN*, *SYNE1*, *MUC16*, *FAT4*, and *ZFHX4* genes showed lower mutation frequencies in our CRC cohort (Figure 3b).

Gene lists with significantly different mutational frequencies between our cohort and the applied reference datasets were determined (Table 2). Altogether, 34 and 45 genes were found in the comparison of CRC samples from Hungary with COCA and TCGA, respectively (*p* < 0.01). The full list can be found in Table S2.

### 3.4. Somatic Mutation Landscape of Colorectal Tumors from a Hungarian Cohort

**Figure 4:**
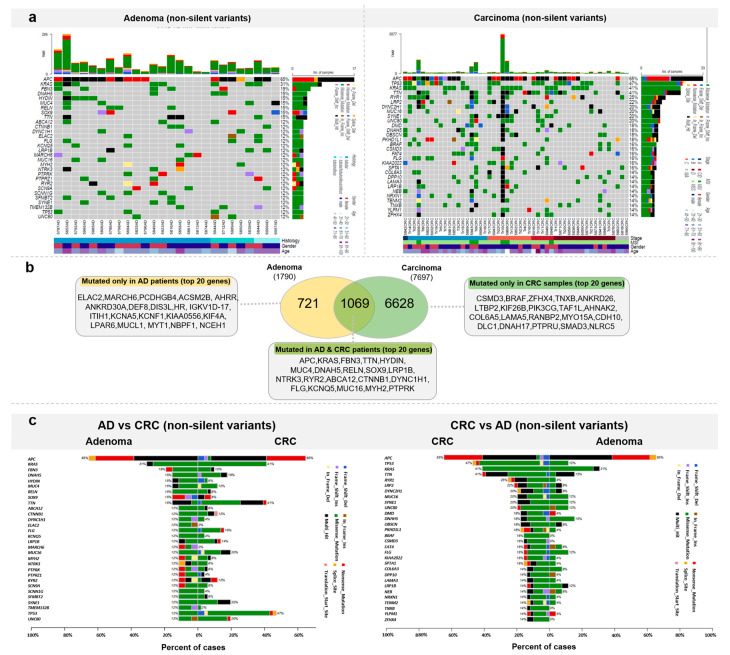
Somatic mutation landscape of colorectal tumors. (**a**) Most frequently mutated genes in adenomas and CRCs (**b**) Venn diagram representation of the number of genes mutated exclusively in one sample group or in both ADs and CRCs with the list of the 20 most frequently mutated genes. (**c**) Direct comparison of AD and CRC samples based on the top 20 most frequently mutated genes in AD (left) and CRC (right).

To explore the somatic landscape of colorectal tumor samples from our cohort, WES was performed on adenomas and CRCs compared with the normal adjacent tissue specimens (Figure 4a).

Altogether, 1790 genes were mutated in the ADs, among which 721 genes were mutated only in this patient group. The top 20 genes mutated in the adenoma patients, but not identified in the CRC most frequently mutated genes, were the following: *ELAC2* and *MARCH6* in 3/27 of AD patients, and *PCDHGB4*, *ACSM2B*, *AHRR*, *ANKRD30A*, *DEF8*, *DIS3L*, *HR*, *IGKV1D-17*, *ITIH1*, *KCNA5*, *KCNF1*, *KIAA0556*, *KIF4A*, *LPAR6*, *MUCL1*, *MYT1*, *NBPF1*, and *NCEH1* in 2/27 of AD patients (Figure 4b).

In the group of genes (1069) found to be mutated both in adenoma and carcinoma samples, the most frequently mutated genes were *APC* (AD: 17/27, CRC: 33/51 (0.647)) and *KRAS* (AD: 8/27, CRC: 21/51 (0.412)). These genes were followed by *FBN3*, *TTN*, *HYDIN*, *MUC4*, *DNAH5*, *RELN*, *SOX9*, *LRP1B*, *NTRK3*, *RYR2*, *ABCA12*, *CTNNB1*, *DYNC1H1*, *FLG*, *KCNQ5*, *MUC16*, *MYH2*, and *PTPRK* (Figure 4b).

In the CRC samples, variants were detected on 7697 genes. Among these, 6628 were characteristic only to CRC patients. The 20 most frequently mutated genes were *CSMD3*, *BRAF*, *ZFHX4*, *TNXB*, *ANKRD26*, *LTBP2*, *KIF26B*, *PIK3CG*, *TAF1L*, *AHNAK2*, *COL6A5*, *LAMA5*, *RANBP2*, *MYO15A*, *CDH10*, *DLC1*, *DNAH17*, *PTPRU*, *SMAD3*, and *NLRC5*. The full list of genes with non-silent mutations detected in AD and CRC can be found in Table S1.

The most frequently mutated genes were compared between the AD and CRC groups. In cases where top AD genes were assessed in the CRC group, *KRAS*, *TTN*, and *TP53* genes were mutated in more CRCs patients than ADs, while *ELAC2*, *MARCH6*, and *TMEM132B* genes were mutated in more AD patients than in CRCs, with a minimum of 10% difference. In the opposite comparison, *TP53*, *KRAS*, *TTN*, *RYR1*, *CRP2*, *DYNC2H1*, *DMD*, *OBSCN*, *PKHD1L1*, *BRAF*, *CSMD3*, *KIA2022*, *SPTA1*, *DPP10*, *LAMA3*, *NRXN1*, *TENM2*, *TNXB*, *YLPM1*, and *FHX4* were more frequently mutated in CRCs than in ADs, with a minimum of 10% difference (Figure 4c). According to Fisher’s exact test, there were seven genes showing significantly (*p* < 0.05) different mutational frequencies between AD and CRC groups (*TP53*, *RYR1*, *ELAC2*, *MARCH6*, *TTN*, *BRAF*, *CSMD3*). The detailed list of differentially mutated genes, *p*-values, and odds ratios are shown in Table S2.

### 3.5. KRAS Mutation Landscape

**Figure 5:**
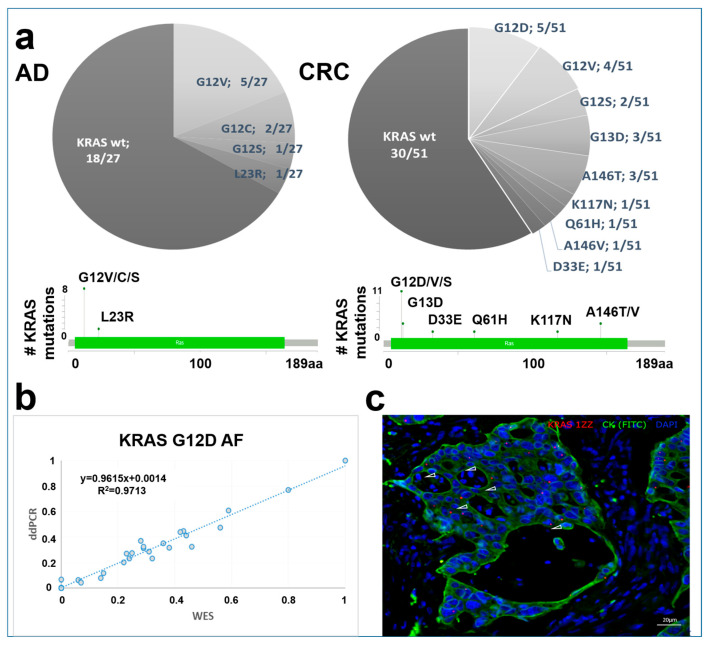
*KRAS* variant distribution in AD and CRC samples. (**a**) *KRAS* variant distribution in AD and CRC groups on pie charts and lollipop plots. (**b**) *KRAS* G12D variant validation using the ddPCR method. The *X*-axis represents WES, while the *Y*-axis shows ddPCR allele frequency (AF) data. (**c**) Representative image of in situ hybridization of *KRAS* G12D mutation in CRC samples. Combined fluorescent image of *KRAS* G12D probes (red, white arrowheads) in CK-positive (green) colon epithelial cells. Nuclei were stained with DAPI.

Among 27 ADs and 51 CRCs, KRAS mutation could be detected in 8/27 and 21/51 (0.41) of the analyzed patients, respectively. *KRAS* variant distribution was different in the case of benign and carcinoma samples. Advanced ADs (with size ≥10 mm and/or high-grade dysplasia and/or villous structure) showed *KRAS* mutation in 6/13 of the cases compared to 2/14 of early ADs. Overall variants in codon 12 (detected in AD:8/27, CRC: 11/51 (0.216) patients) and 13 (CRC: 3/51 (0.06)) were the most frequent in our samples with G12V (5/27 patients) dominance in adenomas and G12D (5/51 (0.098) patients) in CRC cases. Mutations outside of these codons were observed in adenomas (L23R 1/27 patients) and with a higher presence in CRC cases (A146T, K117N, Q61H, A146V, D33E 1/51 (0.019) patients) (Figure 5a).

### 3.6. ddPCR and In Situ Hybridization Validation of KRAS G12D

The most frequently detected *KRAS* variant in the CRC group of the present study, *KRAS* G12D, was assessed by ddPCR to validate our WES allele frequency results. This showed a high correlation between the allele frequencies detected with two independent techniques (R2 = 0.9713) (Figure 5b).

In addition, the *KRAS* G12D mutation was visualized on tissue sections of CRC cases by in situ hybridization. As a confirmation, mutant cases showed *KRAS* G12D signals in the CK-positive epithelial cells (Figure 5c).

### 3.7. Whole Exome Sequencing of cfDNA Samples

**Figure 6:**
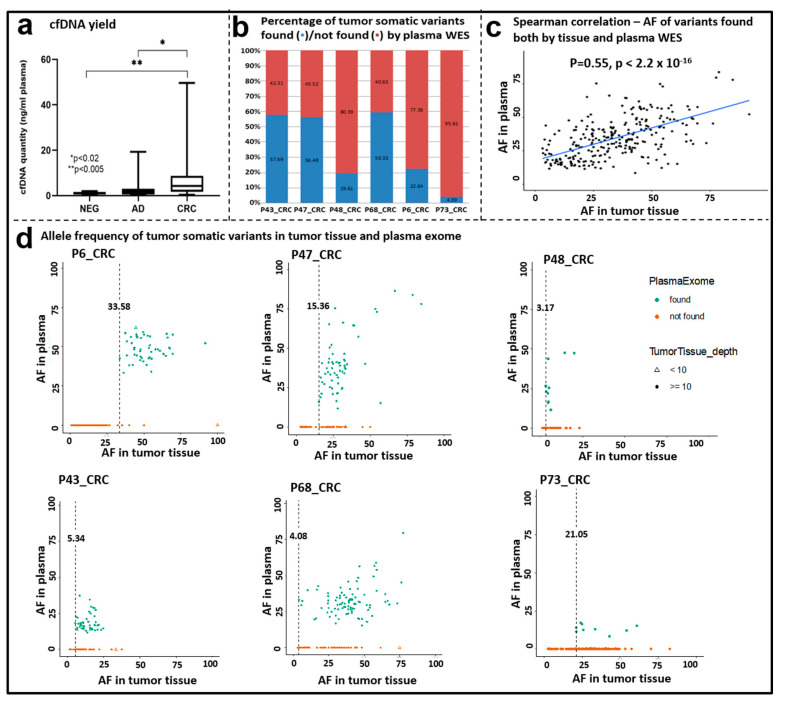
Comparison of tissue and plasma exome sequencing data. (**a**) cfDNA quantity (ng/mL plasma) in the NEG, AD, and CRC groups. Boxplot whiskers represent 2.5–97.5 percentiles, and the center line represents the median. * *p* < 0.02, ** *p* < 0.005. (**b**) Percentage of tumor somatic variants found/not found by plasma cfDNA WES. (**c**) Correlation of the allele frequency data of somatic variants detected both in tissue and plasma samples by WES. Spearman correlation ρ = 0.55, *p* < 2.2 × 10^−16^. (**d**) Allele frequency of variants detected in tissue and plasma samples; dots (•) represent variants with ≥10 coverage; triangles (∆) represent variants <10 coverage in the tissue samples.

Altogether, 57 matched liquid biopsy samples were available with an appropriate amount of cfDNA for exome sequencing (Table 1). To assess the relationship between cfDNA level and tumor progression, plasma cfDNA amounts were quantified. CRC patients had significantly higher cfDNA levels (mean CRC = 8.107 ± 11.46 ng cfDNA/mL plasma) compared to the AD (*p* < 0.02) and N (*p* < 0.005) patients (Figure 6a). The highest plasma cfDNA concentrations were noticed in advanced-stage patients above the age of 60 years.

To explore to what extent somatic variants can be detected in cfDNA samples, WES was performed with a target region of approx. 33 Mb, and 188× mean target coverage depth was achieved. A total of 6 out of 57 patients showed overlapping variants between their matching tissue and cfDNA samples. The percentage of tumor somatic variants observed both in the cfDNA and in the corresponding tissue ranged between 4.39 and 59.35% (Figure 6b). Among the overlapping variants that occurred both in the tumor tissue and in the cfDNA samples, the most abundant variant class was missense mutations, and the most frequent variant types were SNPs followed by DELs. Further classifying SNVs showed that C > T and C > A SNV classes were the most abundant ones (Figure S1). A significant positive correlation was observed between the allele frequency of the variants detected in the tumor tissue and the plasma exome results (Spearman correlation, ρ = 0.55, *p* < 2.2 × 10^−16^) (Figure 6c).

In order to assess if variant detection in plasma cfDNA depends on the allele frequency (AF) of variants in the tumor tissue, the observed AFs in the two sample types were compared. The minimum AF in the tumor tissue when a somatic variant could be detected also in the matching cfDNA sample was 3.17 (P48CRC). This value ranged from 3.17 to 33.58% among the analyzed cohort. On the other hand, remarkable percentages of the variants found in the tissue samples were not detected in the cfDNA samples (Figure 6d).

### 3.8. CRC-Specific Targeted Sequencing

A subset of the cfDNA samples with adequate quantity (30 ng) was also analyzed by a focused targeted panel sequencing approach with a higher sequencing coverage with a solution developed by QIAGEN for reliable calling of low-frequency variants. By using this cfDNA analysis, the tumor somatic variants identified in the tissue samples were found in 8 out of the 12 enrolled patients. Two variants identified with panel sequencing were not targeted by the plasma WES (ERBB SNV C > A, deletion TGC). One patient (P48) did not bear any somatic mutations in the regions targeted by the panel sequencing. The panel sequencing method was able to identify 12/20 variants of all tumor somatic variants covered falling on its targeted regions, while whole-exome sequencing of cfDNA from the same patients recovered only 20% of the tumor somatic variants in the respective regions.

### 3.9. Comparison of WES and Targeted Panel Sequencing Results

**Figure 7:**
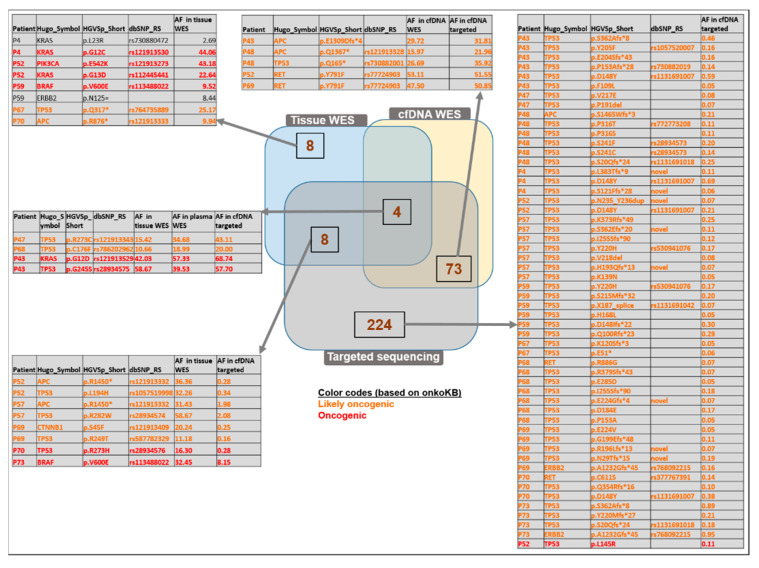
Comparison of the identified variants analyzed with WES of tissue, WES of matched plasma, and targeted sequencing methods. Venn diagram representation defines the number of variants identified commonly or exclusively found with the above-mentioned applied methods. Only the “likely oncogenic” or “oncogenic” variants are listed among the targeted-panel-specific variants and among those detected by both cfDNA WES and targeted panel sequencing. The complete lists can be found in Table S3.

Out of the 20 variants in the target region of the panel sequencing detected either by tissue or cfDNA WES or panel sequencing, there were 4 variants that could be observed with all three methods. Among these variants, two were oncogenic (*KRAS* G12D: 15.42%, 34.68%, and 43.11%; *TP53* G245S: 10.66%, 18.99%, and 20% allele frequencies, respectively) and two were likely oncogenic (*TP53* R273C: 42.03%, 57.33%, and 68.74%; *TP53* C176F: 58.67%, 39.53%, and 57.70% allele frequencies in WES tissue, WES cfDNA, and targeted sequencing, respectively) (Figure 7, Table S3).

Tissue WES and cfDNA targeted sequencing revealed altogether eight variants in common (oncogenic: *BRAF* V600E, TP53 R273H; likely oncogenic: *APC* p.R1450* (detected in two patients), *TP53* p.L194H, *TP53* p.R282W, *CTNNB1* p.S45F, *TP53* p.R249T).

Among the 224 variants exclusively observed by the targeted panel method, 53 likely oncogenic and one oncogenic variants (*TP53* p.L145R) were identified. Most of the 73 variants detected in plasma with both WES and targeted panel sequencing were found also in the normal tissue samples; therefore, these might potentially be germline variants. Interestingly, a subset of variants was observed only in the cfDNA samples but not in the tissue samples (three likely oncogenic: *APC* p.E1309Dfs*, *APC* p.Q1367*, TP53 p.Q165*; one likely neutral: *ERBB2* p.P1170A; Figure 7, intersection of the cfDNA WES and the targeted sequencing results).

## 4. Discussion

Colorectal cancer is one of the leading highly lethal cancer types in Hungary, considered as an epidemiologic challenge that needs to be addressed and explored. To date, an emerging number of CRC-related NGS data are available; however, the majority of the studies originate from the Western population. Therefore, it is necessary to characterize the somatic mutation landscape from Eastern Europeans, e.g., Hungarian patients, with colorectal tumors to find CRC-associated variants with predictive and prognostic potential. On the other hand, there are less identified variants associated with adenomas; however, whole-exome sequencing can reveal novel mutations, even outside of the well-known hot-spot regions that can also have diagnostic, as well as therapeutic values [36].

To investigate the genetic characteristics of colorectal adenomas and cancers from the Hungarian population, firstly, COSMIC mutational signature analysis [37] was performed. This revealed SBS1 and SBS18 as signatures characteristic of AD. SBS1 is considered a nonspecific signature that is characterized by C > T transitions mainly caused by spontaneous deamination of 5 mC, which was also found in MSH3-deficient adenomas by Perne et al. [38]. On the other hand, SBS18 occurs possibly due to damage by reactive oxygen species [39]. CRC samples of our cohort were found to be comparable to the TCGA COAD + READ dataset, as the identified mutation signatures were similar to SBS1, SBS6, and SBS10b. SBS6 is associated with defective DNA mismatch repair that was also found in CRC patients with a familiar history of CRC cases [40]; SBS10b signature primarily occurs in colorectal and uterine cancers [41] and is associated with polymerase epsilon exonuclease domain mutations.

On the basis of the current literature data, a combined analysis of TMB and MSI status can be informative for immune response predictions with clinical relevance [42], as TMB can be utilized as a stratifying marker within MSI-H mCRC for the likelihood of response to immune checkpoint inhibitors [43]. As expected, our adenoma samples showed significantly lower TMB value compared to CRC cases. The difference in the somatic mutation count/Mb between the MSS vs. MSI CRC cases in our cohort was similar to the results of Schrock et al. with 3.5 mutations/Mb (range 0–871) in MSS CRC cases and 46.5 mutations/Mb in MSI-H cases [43].

With exome sequencing, different allele frequencies of somatic mutations in CRC cases from the analyzed Hungarian cohort were compared to both the internationally used TCGA and a Chinese colorectal dataset. A set of genes were identified with significantly different mutational frequencies compared to the reference sets, and 30 genes (including *MUC12*, *ZNF729*, *ENTPD5*, *TRM49B*, and *BCL2*) were only mutated in our cohort but not in the TCGA COAD + READ dataset. Lower expression of mucin 12 (*MUC12*) was found in CRCs in comparison with normal colon samples [44], https://paperpile.com/c/ORDdnG/8xPn, accessed on 22 September 2022, and it was associated with a poorer disease-free survival rate [45]. Zinc finger protein 279 (*ZNF279*) is involved in transcriptional regulation; in the gastrointestinal tract, according to Lin et al., goblet cell adenocarcinomas and intestinal adenocarcinomas cohesive signet ring cell component also harbor mutation in *ZNF279* [46]. Ectonucleoside triphosphate diphosphohydrolase 5 (*ENTPD5*) and *BCL2* are proto-oncogenes, the former showing lower expression in colorectal cancer than in normal mucosa [47], while BCL2 is known to inhibit apoptosis and probably plays a role is the early phases of the adenocarcinoma development [21].

The present work summarizes the first efforts to comprehensively characterize benign adenomatous tissue samples parallel with carcinoma samples from the Hungarian population. Altogether, seven genes were found to show significantly different mutation frequency between ADs and CRCs. In line with current literature data, *TP53* was found to be mutated with significantly higher frequency among CRC cases than in adenomas, leading to the inactivation of this gene and contributing to the transition step from adenoma to carcinoma [12,48]. The gene encoding ryanodine receptor 1 (*RYR1*)—identified as a mutated gene in CRCs from the Thai population compared to the matched normal samples, but not in adenomas [49]—was also predominantly mutated in our CRC group. We identified two genes (*ELAC2*, *MARCH6*) with significantly higher mutation frequency in ADs than in CRCs. ElaC ribonuclease Z 2 (*ELAC2*) is primarily associated with prostate cancer [50], and *MARCH6*, along with *ACSM2B*, *DEF8*, *DIS3L*, and *KCNA5* gene variants, have not been associated with colonic adenomas yet. In the comparative study of Wolff et al., compared to our samples, *TTN* was found to be mutated in a higher frequency in Ads (7/17 vs. our ADs: 4/27) and also in MSS carcinomas (8/16 vs. our CRCs: 21/51) [51]. Mutated BRAF gene is an oncogenic event, an independent prognostic factor, and can be found in about 10% of CRC patients [52,53], while we detected it in 8/51 CRC cases, but not in ADs. Yi et al. found mutated BRAF in 22/48 analyzed intraepithelial neoplasias from the Chinese population [54]. *CSMD3* was found to be mutated in 30/148 CRC patients documented by Wolff et al. [51], while it was altered with slightly lower frequency, in 8/51 CRCs in our cohort, and it was included in a 20-gene panel that can distinguish colorectal adenoma from adenocarcinoma, established by Lin et al. [55]. Among the mutated genes in the adenomatous tissue samples involved in our study, *PCDHGB4* [56], *AHRR* [57], *KIF4* [58,59], *LPAR6* [22,60], *NBPF1* [61], and *NCEH1* [62] were already associated with colorectal cancer formation, while *ANKRD30A* [63], *ITIH1* [64], and *KIAA0556* [65] were related to other types of cancers. Interestingly, *NBPF1*—mutated in 2/27 of our adenoma samples—is a cancer driver gene in CRC as a tumor suppressor and also a potential regulator and biomarker for CRC [66].

*KRAS* is one of the most explored genes frequently mutated in colorectal neoplasia; its mutation frequency varies from 20% to 70% in colorectal adenomas [67], and approx. 40–52% of CRC cases bear *KRAS* mutation [68]. It holds a great clinical impact, as these variants are associated with poor prognosis and drug resistance [69]. In our cohort, 9/27 of ADs showed *KRAS* mutation, predominantly in advanced adenomas. In the study of Yadamsuren et al., also analyzing Hungarian patients, 49.4% of the adenomas had *KRAS* mutation (57.5% of advanced adenomas, 31.0% of non-advanced adenomas) [67], while Juarez et al. found mutated *KRAS* in 11.6% of patients with conventional adenomas from a Spanish cohort [23]. It is known that *KRAS* mutation frequency shows considerable variability in CRC cases; for instance, it is associated with different ethnicities (Caucasians: 38%; Asians: approx. 40%; Africans: 21%) [70]. According to Phipps et al., among 1989 cases, 31% had KRAS-mutated CRC [71], while in our Hungarian cohort, 21/51 of CRCs were KRAS-mutated, as detected by tissue WES. Upon the analysis of 56 CRC cases, Roa et al. found slightly higher frequencies in codons 12 and 13 (G12D 39.1%, G12V 24.2%, G12S 6.5%) compared to our cohort and also found mutations in codons that we have not observed [72]. Our WES *KRAS* G12D results were confirmed by two independent methods. Firstly, ddPCR revealed high concordance with similar allele frequency results to those obtained by tissue WES; furthermore, as an in situ validation, *KRAS* G12D mRNAs were also recognized in the tissue sections of the identified mutant CRC cases. Mutations aside from the hotspot sites also hold clinical importance. For instance, variants in codon A146T—detected in 3/51 of our CRC cases—promote EGFR resistance; however, these are associated with better overall survival compared to codon 12 mutations [24]. A146 mutations were noticed in a combined frequency of 4% in a study analyzing CRC cases from Hong Kong and the USA [73], while in our sample set, this value was 4/51 (A146T, A146V). Similar to our results, Edkins et al. found Q61 mutations in 2% of CRCs [73].

Several studies have addressed the potential utility of cfDNA in CRC diagnosis [25,74,75,76], as it can fulfill the promise of a minimally invasive diagnostic approach that overcomes the issue of tumor heterogeneity [19]. The quantitative cfDNA analysis can be applied as a good marker since its appearance in blood is an indicator for adverse RFS and OS in CRC patients [77]. In the present study, we observed a significant cfDNA amount elevation in AD and CRC groups compared to healthy cases.

Our WES plasma results showed that 6/57 of our patients had overlapping variants with their corresponding tissue samples, and 4.4–59.4% of the tumor somatic variants could be found. These results are in line with literature data, as variable concordance was observed in similar studies between matched tissue and cfDNA-based analyses. In a meta-analysis by Bos et al., the overall agreement between cfDNA and matched tumor tissue was found to be 31% (shared/all SNV*100%) [78]. Regarding the detected allele frequency data, as a confirmation of the accurate detectability of the variants in the cfDNA samples, we observed significantly high concordance in the matched gDNA–cfDNA comparisons in the case of the overlapping variants. In the study of Diefenbach et al., metastatic melanoma patients showed similar variety in the degree of overlap between tissue- and plasma-derived data (ranging from 22.7% to 77.6%) [79]. Conversely, others, e.g., Ju et al., found moderate positive correlations in mutant allele frequencies of variants shared between tissue and plasma in endometrial cancer cases [80]. In the literature, diverse concordance rates are reported between mutations detected in tissue or cfDNA in CRC patients. The main potential factors in the background of lacking concordance include the different sample processing procedure and assays, spatial tumor heterogeneity, and the presence of subclones [81]. In a comparative study of Liebs et al., CRC was found to show the best concordance (63%) in the mutation profile between tissue and cfDNA samples, followed by melanoma and HNSCC (55% and 11%, respectively), possibly due to the different heterogeneity levels of solid tumors [25]. In contrast, Lebofsky et al. reported 79% matching mutations in cfDNA and tumor biopsies from metastatic cancer patients, recovering 28 of 29 (97%) tissue mutations in plasma [82]. Different groups reported that 56–87% of tissue mutations could be detected in the plasma of CRC patients [25,83,84,85]. On the other hand, explanation behind the mutations found exclusively in liquid biopsies can be the possible presence of multiple cancer foci [25], which might not be totally covered by tissue biopsy sampling. Therefore, it is always important to take tumor heterogeneity into account, and the comparison of multiple tissue biopsies with the corresponding liquid biopsy might be advantageous.

Taken together, clinically relevant variants, such as *KRAS* G12D mutation, were detectable by tissue, plasma WES, and the targeted panel sequencing in our cohort. These results highlight that WES of cfDNA can still be considered as a feasible alternative of tissue biopsies detecting genetic variants; however, panel sequencing has a higher potential of detecting alterations with lower frequency. The targeted analysis of CRC-related genes and hotspots revealed several oncogenic and likely oncogenic variants, and approximately one-third of them overlapped with plasma WES with similar MAF values. A group of variants was only detected with the targeted panel, and with a relatively low allele frequency; in their case, we cannot exclude the possibility that these are false positives. The filtering strategy of non-tumor derived cancer-like genomic alterations remains to be a challenge [86].

In the combined comparison of the tissue, plasma WES, and the targeted panel, four likely oncogenic and oncogenic variants (*TP53* p.C176F, *KRAS* p.G12D, *TP53* p.G245S, *TP53* p.R273C) were detected. Among them, the first three were already associated with colon cancer; *TP53* p.C176F was detected in CRC patients from the Taiwanese population [87], while *TP53* p.G245 variants were associated with a significantly increased risk of death due to colon cancer [88]. The *TP53* p.R273C variant was associated with tumor cell growth in lung cancer and poor prognosis in low-grade glioma patients [89,90].

Besides genetic features, in the future, it would be also favorable to identify other regulatory factors, gaining more insight into the complex picture of the molecular background of the Hungarian CRC cases. Furthermore, the conscious modification of lifestyle would certainly reduce the burden of CRC cases in Hungary.

One of the strengths of the present study is the comprehensive analysis workflow applied to Hungarian patients that can be a valuable source for an Eastern European database. Moreover, certain limitations should be also considered, as our single-center sample collection resulted in low sample numbers with limited metadata, and in parallel, the number of cfDNA samples analyzed with targeted sequencing needs to be expanded to better understand the genetics accessible from the plasma of patients with colorectal lesions. Further research is required to include more patients from other centers nationwide, providing a more detailed description of the variants of Hungarian patients.

## 5. Conclusions

TMB value was found to be slightly higher in our analyzed cohort than the Chinese cohort, while it was similar to the TCGA COAD + READ datasets. The somatic mutation landscape of benign and malignant colon lesions showed distinct patterns, while most of the identified variants were common in Ads and CRCs. Among *KRAS* variants, codons 12 and 13 were the most frequent in our samples, with G12V dominance in adenomas and G12D in CRC cases. This latter, clinically relevant variant was identified at the tissue level with ISH in the corresponding samples. According to our WES results, a high correlation was found between matched tissue and plasma variant allele frequencies. Panel sequencing with a higher coverage revealed 12/20 of all tumor somatic variants, falling on its targeted regions, while WES recovered only 20% in the respective regions in cfDNA of the same patients. Taken together, in the case of liquid biopsy analyses, WES is less efficient compared to the targeted panel sequencing with a higher coverage depth that can hold a relevant clinical potential in the future in order to be applied in everyday practice.

## Figures and Tables

**Table 1:** Clinicopathological characteristics of patients analyzed in the current study (N—healthy, AD—adenoma, CRC—colorectal cancer patient).

Characteristics		Tissue WES	Plasma WES	Plasma Targeted Panel
Age		*n* (Years, (Median))	*n* (Years, (Median))	*n* (Years, (Median))
	N	18 [21]	7 [22]	
	AD	27 [23]	17 [23]	
	CRC	51 [24]	33 [69.5]	11 [25]
Sex		*n* = 92	*n* = 57	*n* = 11
	Male	48	29	7
	Female	44	28	4
Anatomic location of the primary lesion				
AD		*n* = 27	*n* = 17	
	Cecum	1	1	
	Ascending colon	1		
	Descending colon	1	1	
	Sigmoid colon	6	3	
	Rectosigmoid	4		
	Rectum	2	1	
	Multiple locations	12	11	
CRC		*n* = 51	*n* = 33	*n* = 11
	Cecum	10	6	2
	Ascending colon	5	5	2
	Transverse colon	3	4	2
	Descending colon	1		
	Sigmoid colon	11	8	2
	Rectosigmoid	2		1
	Rectum	16	7	2
	Multiple location	6	3	1
Adenoma histology		*n* = 27	*n* = 17	
	Tubular	19	13	
	Tubulovillous	5	3	
	Tubular–tubulovillous	2	1	
CRC AJCC stage		*n* = 51	*n* = 33	*n* = 11
	I	5	5	1
	lla	7	8	2
	llb	1	1	
	llla	9	2	
	IIIb	5	3	
	lllc	4	4	1
	IV	17	10	7
	Unkown	3		

**Table 2:** Fisher’s exact test results of the comparison of CRCs from our cohort with COCA and TCGA (*p* < 0.01).

Hugo_Symbol	SE-CRC	COCA	*p*-Value	Odds Ratio
*ZNF717*	1	73	5.70 × 10^−5^	6.11 × 10^−2^
*FRG1*	0	55	1.06 × 10^−4^	0.00 × 10^0^
*MUC3A*	0	51	1.93 × 10^−4^	0
*MUC6*	3	82	3.43 × 10^−4^	1.63 × 10^−1^
*KIAA2022*	8	7	3.76 × 10^−4^	7.58 × 10^0^
*DBF4*	4	0	4.26 × 10^−4^	Inf
*KCNJ12*	0	46	5.57 × 10^−4^	0
*ZSCAN5A*	5	2	9.68 × 10^−4^	1.57 × 10^1^
*FNDC3A*	4	1	1.90 × 10^−3^	2.46 × 10^1^
*ITGB2*	4	1	1.90 × 10^−3^	2.46 × 10^1^
*OR1M1*	4	1	1.90 × 10^−3^	2.46 × 10^1^
*SMAD3*	6	5	2.00 × 10^−3^	7.66 × 10^0^
*CCDC171*	5	3	2.29 × 10^−3^	1.05 × 10^1^
*LTBP2*	7	8	2.56 × 10^−3^	5.66 × 10^0^
*MUC2*	2	61	2.69 × 10^−3^	1.57 × 10^−1^
*RP11-764K9.4*	0	39	2.82 × 10^−3^	0
*ALG11*	3	0	3.04 × 10^−3^	Inf
*CCDC86*	3	0	3.04 × 10^−3^	Inf
*OC90*	3	0	3.04 × 10^−3^	Inf
*PRKAR1B*	3	0	3.04 × 10^−3^	Inf
*SLC25A44*	3	0	3.04 × 10^−3^	Inf
*UBOX5*	3	0	3.04 × 10^−3^	Inf
*DYNC2H1*	10	18	3.30 × 10^−3^	3.73 × 10^0^
*HLA-DRB1*	0	34	4.59 × 10^−3^	0
*ALDH3A2*	4	2	5.06 × 10^−3^	1.23 × 10^1^
*ALPL*	4	2	5.06 × 10^−3^	1.23 × 10^1^
*MFN2*	4	2	5.06 × 10^−3^	1.23 × 10^1^
*SEMG2*	4	2	5.06 × 10^−3^	1.23 × 10^1^
*PTPRU*	6	7	5.84 × 10^−3^	5.44 × 10^0^
*RANBP2*	6	7	5.84 × 10^−3^	5.44 × 10^0^
*DPP10*	7	10	6.07 × 10^−3^	4.50 × 10^0^
*FRG2C*	0	32	7.50 × 10^−3^	0
*FRG1B*	0	31	7.66 × 10^−3^	0
*TNXB*	7	11	8.81 × 10^−3^	4.08 × 10^0^
***Hugo_Symbol***	**SE-CRC**	**TCGA**	***p*-Value**	**Odds Ratio**
*MUC12*	4	0	5.10 × 10^−5^	Inf
*ZNF729*	4	0	5.10 × 10^−5^	Inf
*ENTPD5*	3	0	6.21 × 10^−4^	Inf
*TRIM49B*	3	0	6.21 × 10^−4^	Inf
*BCL2*	4	2	6.70 × 10^−4^	2.24 × 10^1^
*B4GALNT3*	5	5	7.38 × 10^−4^	1.14 × 10^1^
*NMBR*	5	5	7.38 × 10^−4^	1.14 × 10^1^
*FOXD3*	4	3	1.46 × 10^−3^	1.50 × 10^1^
*SLC7A7*	4	3	1.46 × 10^−3^	1.50 × 10^1^
*ZSCAN5A*	5	9	4.47 × 10^−3^	6.33 × 10^0^
*ALDH3A2*	4	5	4.61 × 10^−3^	8.97 × 10^0^
*PIK3CA*	4	132	5.00 × 10^−3^	2.61 × 10^−1^
*C1orf43*	3	2	5.47 × 10^−3^	1.65 × 10^1^
*CDT1*	3	2	5.47 × 10^−3^	1.65 × 10^1^
*GPR27*	3	2	5.47 × 10^−3^	1.65 × 10^1^
*KRTAP13-4*	3	2	5.47 × 10^−3^	1.65 × 10^1^
*STMN2*	3	2	5.47 × 10^−3^	1.65 × 10^1^
*ZFHX2*	3	2	5.47 × 10^−3^	1.65 × 10^1^
*SHCBP1L*	5	10	6.26 × 10^−3^	5.69 × 10^0^
*AL033381.1*	2	0	7.41 × 10^−3^	Inf
*ANHX*	2	0	7.41 × 10^−3^	Inf
*ANKRD31*	2	0	7.41 × 10^−3^	Inf
*C11orf85*	2	0	7.41 × 10^−3^	Inf
*ERVMER34-1*	2	0	7.41 × 10^−3^	Inf
*ERVV-1*	2	0	7.41 × 10^−3^	Inf
*FAM153C*	2	0	7.41 × 10^−3^	Inf
*FAM188B2*	2	0	7.41 × 10^−3^	Inf
*FBXL8*	2	0	7.41 × 10^−3^	Inf
*FREM3*	2	0	7.41 × 10^−3^	Inf
*GCGR*	2	0	7.41 × 10^−3^	Inf
*IZUMO3*	2	0	7.41 × 10^−3^	Inf
*KRTAP9-1*	2	0	7.41 × 10^−3^	Inf
*LRRC53*	2	0	7.41 × 10^−3^	Inf
*MUC22*	2	0	7.41 × 10^−3^	Inf
*OR56B3P*	2	0	7.41 × 10^−3^	Inf
*PP2D1*	2	0	7.41 × 10^−3^	Inf
*RP11-10J21.3*	2	0	7.41 × 10^−3^	Inf
*RP11-766F14.2*	2	0	7.41 × 10^−3^	Inf
*SAR1B*	2	0	7.41 × 10^−3^	Inf
*STARD9*	2	0	7.41 × 10^−3^	Inf
*TDRD15*	2	0	7.41 × 10^−3^	Inf
*TRAV9-1*	2	0	7.41 × 10^−3^	Inf
*TRIM64C*	2	0	7.41 × 10^−3^	Inf
*YRDC*	2	0	7.41 × 10^−3^	Inf
*ZSCAN5C*	2	0	7.41 × 10^−3^	Inf

## Data Availability

The study was conducted in the framework of the Hungarian Oncogenome Program, and the data are available at https://cbioportal.vo.elte.hu/cbioportal; accessed on 19 September 2022.

## Supplementary Materials

The following supporting information can be downloaded at https://www.mdpi.com/article/10.3390/cancers15030907/s1. Table S1: Full list of non-silent mutations identified in AD and CRC groups by tissue WES (Table S1.xls). Figure S1: Variant classification of the variants detected in both tissue and plasma WES experiments (Figure S1.tif). Table S2: Lists of differentially mutated genes in the comparison of AD vs. CRC, CRC vs. TCGA, and CRC vs. COCA, including *p*-values and odds ratios. Table S3: Table of the overlapping variants covered by the targeted panel regions in tissue, plasma WES, and targeted panel (Table S3).
