# Supplementary figures and images for: Patterns of Somatic Variants in Colorectal Adenoma and Carcinoma Tissue and Matched Plasma Samples from the Hungarian Oncogenome Program
Source: Cancers (Basel). 2023 Jan 31;15(3):907. doi: 10.3390/cancers15030907 (PMC9913259; doi:10.3390/cancers15030907)

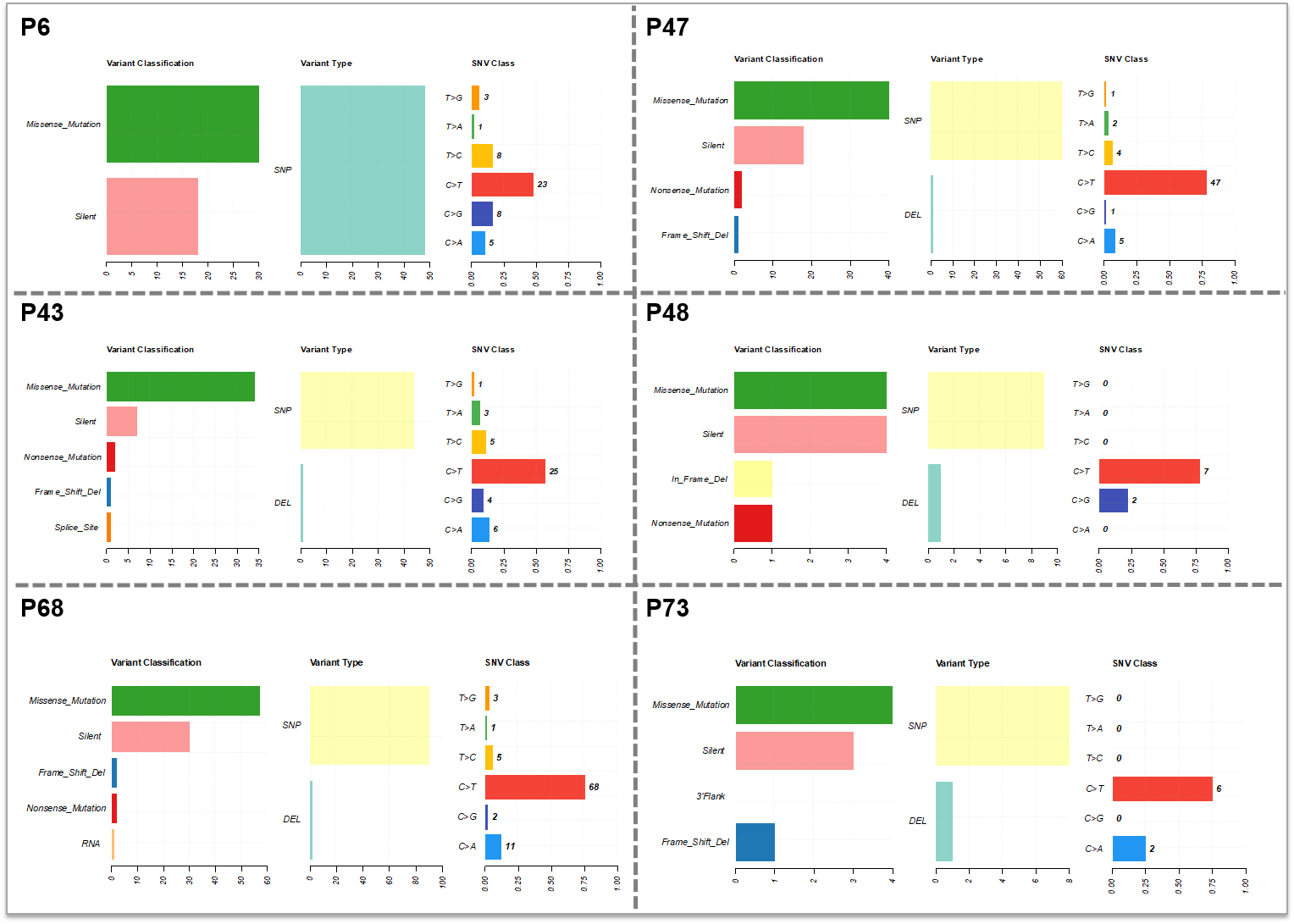

Supplement: Supplementary file 1 [file cancers-15-00907-s001.zip › Figure S1.tif]
